# Dementia as an Independent Predictor of Falls in Older Breast Cancer Survivors: Evidence From a Real World Multicenter Electronic Health Record Network

**DOI:** 10.21203/rs.3.rs-8124537/v1

**Published:** 2025-11-28

**Authors:** Asmaa Namoos, Oxana Palesh, Nicholas Thomson, Faika Zanjani

**Affiliations:** Virginia Commonwealth University; Virginia Commonwealth University; Virginia Commonwealth University; Virginia Commonwealth University

**Keywords:** Breast cancer, dementia, falls, survivorship, geriatric oncology, electronic health records, TriNetX, risk prediction, neurological comorbidity, polypharmacy

## Abstract

**Introduction:**

Falls are a major source of morbidity in older adults and pose particular concern in cancer survivors who may experience treatment related neurological and functional decline. Dementia is a known risk factor for falls, yet its contribution to fall risk among breast cancer survivors has not been well defined.

**Methods:**

This retrospective cohort study used de identified electronic health records from the TriNetX Research Network, which includes more than 100 health care organizations. Women aged 65 years or older with stage 1 to stage 3 breast cancer were eligible. Dementia was identified using ICD 10 code F03 recorded on or after the first qualifying cancer diagnosis. Propensity score matching used a 1 to 1 nearest neighbor approach. The primary outcome was incident fall events identified by ICD 10 codes for unspecified falls, initial fall encounters, history of falling, and repeated falls. Multivariable Cox proportional hazards models estimated independent predictors of falls. Follow up began 1 day after diagnosis and continued through the last recorded encounter.

**Results:**

A total of 49 576 breast cancer survivors met inclusion criteria, of whom 1 683 (3.4%) had dementia. Before matching, fall related diagnoses were significantly more common in patients with dementia, including unspecified falls (26% vs 2%, p < 0.0001) and history of falling (15% vs 1%, p < 0.0001). After 1 to 1 matching, 1 602 survivors remained in each cohort with standardized mean differences < 0.06 across all variables. During follow up, 17.8% of survivors with dementia experienced a fall compared with 6.5% without dementia. This corresponded to an absolute risk difference of 11.3% (95% CI 9.1% to 13.6%), a risk ratio of 2.74 (95% CI 2.41 to 3.12), and an odds ratio of 3.12 (95% CI 2.67 to 3.65). The Kaplan Meier analysis showed significantly lower fall free survival in the dementia cohort (log rank p < 0.0001). The adjusted Cox model showed that dementia remained an independent predictor of falls (hazard ratio 1.43, 95% CI 1.25 to 1.63). Additional strong predictors included long term drug therapy (hazard ratio 2.62, 95% CI 2.41 to 2.84), osteoporosis (hazard ratio 1.48, 95% CI 1.34 to 1.62), polyneuropathy (hazard ratio 1.58, 95% CI 1.34 to 1.85), and depressive episode (hazard ratio 1.78, 95% CI 1.60 to 1.98).

**Conclusions and Relevance:**

Dementia was associated with a substantially elevated fall risk among older breast cancer survivors, even after extensive adjustment for comorbidity, neurological conditions, psychiatric disorders, and medication burden. Recognition of this risk may help clinicians identify a subgroup of survivors who require closer monitoring and more precise evaluation during routine care.

## BACKGROUND

Recent research in cancer neuroscience is reshaping our understanding of how tumors and the brain communicate. Cancer is not confined to unchecked cell growth; it interacts with neural circuits and the central nervous system in ways that influence tumor progression, repair, inflammation, and even systemic vulnerability. In parallel, the burdens of cancer treatment often extend beyond the tumor management; treatments affect brain health, cognition, neurological resilience, and physical function^1,2^. In survivors of breast cancer, this “neural legacy” can manifest as subtle to overt cognitive dysfunction, neuropathy, and neurologic comorbidities.

Many breast cancer survivors report persistent cognitive changes such as slower information processing, difficulties with attention or multitasking, and memory lapses^3,4^. This phenomenon, often termed cancer-related cognitive impairment (CRCI), has been documented across many studies, even years after treatment ends^5^. In survivors, factors such as surgical stress, chemotherapy, radiation, endocrine therapy, brain aging, and systemic inflammation may act in concert to weaken neural reserve and plasticity^6^. Moreover, these cognitive changes do not occur in isolation: they often overlap with other treatment sequelae such as peripheral neuropathy, fatigue, sarcopenia, and functional decline^7^.

In older adults, cognitive vulnerability is a known contributor to fall risk^8,9^. Impaired attention, slowed reaction time, reduced capacity to adapt to environmental challenges, and poorer executive control all compromise physical balance and increase the likelihood of missteps or instability^10^. Among patients with dementia or mild cognitive impairment, fall rates are significantly higher compared to peers with normal cognitive function^11^. Yet, little is known about how this risk plays out in the context of cancer survivorship, where multiple intersecting vulnerabilities (e.g., older age, prior treatments, comorbid disease, polypharmacy) coexist.

Breast cancer survivors represent a particularly important population in this regard. Because breast cancer is common and survival rates are high, a growing number of women live long after diagnosis and therapy^12^. As this survivor population ages, neurological and functional health become central to quality of life and independence. However, in this group, the interplay between preexisting or new-onset dementia and fall risk remains largely unexplored. Anecdotally, clinicians observe that older survivors with cognitive impairment may fall more, but published evidence is scarce^13^.

Our study seeks to fill this gap by quantifying the comparative risk of falls among older breast cancer survivors with versus without dementia, using a large real-world electronic health record network. We also aim to disentangle the independent effect of dementia after adjusting for demographic, frailty, neurologic and psychiatric comorbidities, and medication burden. We hypothesize that dementia will remain a robust independent predictor of fall risk, even in this medically complex population. By doing so, we hope to inform fall-prevention strategies tailored for cognitively impaired cancer survivors and guide risk stratification in survivorship care.

## METHODS

Data Source. This retrospective cohort study utilized data from the TriNetX Research Network, a federated platform aggregating de-identified electronic health records from more than 100 health care organizations. TriNetX enables the construction of complex cohort queries based on diagnosis, procedure, demographic, and temporal parameters while maintaining full compliance with HIPAA and GDPR standards. All data are de-identified prior to analysis; therefore, institutional review board approval was not required.

Study Population. Cohorts were identified using ICD-10-CM code C50 (malignant neoplasm of breast) and AJCC stage information from the TriNetX Research Network^14^. Eligible participants were women aged 65 years or older with stage I–III breast cancer and complete follow-up data. Stage 0 cases were excluded because in situ disease rarely leads to treatment-related cognitive or physical decline, while stage IV cases were excluded due to advanced illness and high baseline fall risk. Limiting the sample to stages I–III ensured a comparable survivorship population for evaluating dementia-related fall risk. After applying these criteria, 1,684 dementia cases and 55,837 non-dementia comparators were retained for baseline analyses. Minor differences between the initial and analytic counts reflect automated TriNetX dataquality filters, which exclude records with missing variables or inconsistent temporal relationships between diagnoses, such as dementia documented before the breast cancer diagnosis. At the time of analysis, data were contributed by 109 of 111 participating health care organizations across the network.

Time Window and Index Event. A five-year look-back period (1,825 days) was applied to ensure accurate temporal alignment between dementia and breast cancer diagnoses. The index date was defined as the first recorded breast cancer diagnosis meeting all inclusion criteria. The observation window extended from 1,825 days before to 1 day prior to the index event, ensuring that dementia or comparator diagnoses occurred within a clinically relevant period while excluding cases in which dementia pre-dated the cancer diagnosis.

Dementia Cohort Definition. The dementia cohort included women aged 65 years and older with a documented diagnosis of unspecified dementia (ICD-10-CM F03) recorded on or after the index breast cancer diagnosis. This specification allowed same-day documentation but excluded any dementia diagnosis occurring before cancer diagnosis to capture cognitive decline concurrent with or following breast cancer. The comparison cohort comprised patients who met all other eligibility criteria but had no record of dementia (F03) during the observation period. This temporal structure minimized the risk of reverse causality by ensuring that dementia represented post-diagnostic or concurrent impairment rather than preexisting cognitive decline.

Outcome Definition. The primary outcome was the occurrence of a fall following the breast cancer diagnosis. Fall-related events were defined using ICD-10-CM codes W19 (unspecified fall), W19.XXXA (unspecified fall, initial encounter), Z91.81 (history of falling), and R29.6 (repeated falls). To ensure that only incident falls were captured, patients with documented fall diagnoses prior to the index breast cancer diagnosis were excluded. The index date was defined as the earliest recorded date of breast cancer diagnosis. Patients were followed from one day after the index date until the last available encounter, death, or censoring.

Covariates. Clinical covariates were selected based on prior literature and clinical relevance. These included demographic factors (age, race, and ethnicity), frailty and musculoskeletal conditions (osteoporosis, sarcopenia, cachexia, muscle wasting), neurological disorders (polyneuropathy, Parkinson’s disease, sequelae of cerebral infarction, unsteadiness, and other gait abnormalities), psychiatric conditions (major depressive disorder and depressive episodes), comorbidities (heart failure, malnutrition, pressure ulcer), and medication burden, represented by long-term drug therapy (ICD-10-CM Z79) as a proxy for polypharmacy (Table 1).

Statistical Analysis. Descriptive statistics summarized baseline characteristics for dementia and non-dementia cohorts. Continuous variables were compared using Student’s t-tests, and categorical variables were compared using chi-square tests. Standardized mean differences were calculated to evaluate balance between groups, with a value below 0.1 indicating adequate balance. Propensity score matching (1:1 nearest neighbor) was performed to reduce confounding, and covariate balance was reassessed post-matching. Kaplan-Meier curves were generated to compare fall-free survival between cohorts, and differences were evaluated using the log-rank test. Cox proportional hazards regression models were fitted to estimate the independent association between dementia and risk of falling, adjusting for age, race, comorbidities, frailty, neurologic, and psychiatric conditions, as well as polypharmacy. Hazard ratios (HRs) with 95% confidence intervals (CIs) were reported, and statistical significance was defined as a two-sided p-value < 0.05. All analyses were conducted within the TriNetX analytics environment using its built-in statistical functions.

## RESULTS

Baseline Demographics. Breast cancer survivors with dementia (n = 1,683) differed substantially from those without dementia (n = 48,926) in demographic composition and fall-related health profiles (Table 2). All participants were female and aged 65 years or older. The dementia cohort was significantly older, with a mean current age of 84.3 ± 6.44 years compared with 76.3 ± 7.54 years among survivors without dementia (p < 0.0001). The mean age at index cancer diagnosis was also higher in the dementia group (79.9 ± 7.40 years) than in the non-dementia group (67.2 ± 8.84 years; p < 0.0001). Racial and ethnic distributions varied significantly. Survivors with dementia were more frequently White (66%) or Black/African American (25%) than those without dementia (59% and 11%, respectively; both p < 0.0001). In contrast, the non-dementia cohort included a greater proportion of patients with Unknown race (18% vs 2%) and other race (6% vs 2%). Rates of Asian, Hispanic/Latino, Native Hawaiian/Pacific Islander, and American Indian/Alaska Native race categories were low and did not differ significantly. Similarly, more dementia patients were identified as Not Hispanic or Latino (70% vs 50%, p < 0.0001), whereas Unknown ethnicity was more common in the non-dementia group (46% vs 27%, p < 0.0001). These findings indicated significant baseline heterogeneity between the two cohorts prior to adjustment. (Table 2).

Baseline Fall-Related Diagnoses. Before the index breast cancer diagnosis, fall-related conditions were more frequently documented among survivors with dementia than among those without dementia. Unspecified falls were recorded in 26% (n = 434) of the dementia cohort and in 2% (n = 1,038) of the non-dementia cohort (p < 0.0001; standardized difference 0.73). Initial encounters for unspecified falls occurred in 25% (n = 415) of survivors with dementia and in 2% (n = 980) of those without dementia (p < 0.0001; standardized difference 0.71). The history of falling was present in 15% (n = 259) of the dementia cohort and in 1% (n = 403) of the non-dementia cohort (p < 0.0001; standardized difference 0.55). Repeated falls were recorded in 11% (n = 180) of survivors with dementia compared with less than 1% (n = 191) of those without dementia (p < 0.0001; standardized difference 0.46). Fall-related injuries coded as injury of unspecified body region (T14) were reported in 8% (n = 141) of the dementia cohort and in 1% (n = 576) of the non-dementia cohort (p < 0.0001; standardized difference 0.34) (Table 3).

### Propensity Score Matching

After 1:1 propensity score matching, 1,602 patients were retained in each cohort. Matching achieved excellent balance across all demographic covariates, with standardized mean differences below 0.06 (Table 3). Mean current age was virtually identical between dementia and non-dementia patients (84.1 ± 6.50 vs 84.1 ± 6.56 years, p = 0.98), as was age at index diagnosis (79.5 ± 7.39 vs 79.5 ± 7.52 years, p = 0.99). Racial and ethnic distributions were similarly well balanced after matching. The proportion of White (65.6% vs 65.4%) and Black/African American (24.3% vs 25.5%) patients was nearly identical, and no residual significant differences remained across Asian, Hispanic/Latino, or other racial categories (all p > 0.05). Ethnic proportions also aligned closely (Not Hispanic or Latino 69.7% vs 72.0%; p = 0.15) (Table 4).

Baseline Fall-Related Diagnoses. Prior to propensity score matching, fall-related conditions were markedly more prevalent among breast cancer survivors with dementia compared to those without. Unspecified falls (W19) were documented in 25.8% of the dementia cohort versus 2.1% of the non-dementia cohort (p < 0.0001). Similarly, initial encounters for unspecified falls (W19.XXXA) occurred in 24.7% versus 2.0% of patients, respectively (p < 0.0001). A documented history of falling (Z91.81) was present in 15.4% of dementia patients compared with 0.8% of those without dementia (p < 0.0001). Repeated falls (R29.6) were identified in 10.7% versus 0.4% (p < 0.0001), and injuries of unspecified body region (T14) were more frequent among dementia patients (8.4% vs 1.2%; p < 0.0001).

After 1:1 propensity score matching, these conditions were well balanced across groups. The matched dementia and non-dementia cohorts each included 1,602 patients, with nearly identical frequencies of unspecified falls (22.9% vs 23.1%; p = 0.87), initial encounters for unspecified falls (21.8% vs 22.2%; p = 0.77), history of falling (12.6% vs 11.2%; p = 0.25), repeated falls (8.4% vs 7.0%; p = 0.14), and injury of unspecified body region (7.4% vs 7.9%; p = 0.60). All standardized mean differences were below 0.06, confirming adequate balance in fall-related diagnoses following matching (Table 5).

### Copy/right of the graph to TriNetX Platform

#### Fall Outcomes and Risk Estimates

Measures of Association. After excluding patients with falls prior to the index period (cancer diagnosis), breast cancer survivors with dementia exhibited a significantly higher risk of subsequent falls compared with those without dementia. Among the dementia cohort, 17.8% (202 of 1,134) experienced a fall during follow-up compared with 6.5% (3,096 of 47,639) in the non-dementia cohort. The absolute risk difference was 11.3% (95% CI 9.1–13.6, p < 0.0001), while the relative risk was 2.74 (95% CI 2.41–3.12). Similarly, the odds of falling were more than three times higher in the dementia group compared to the non-dementia group (odds ratio 3.12, 95% CI 2.67–3.65) (Table 6).

**Figure 2:**
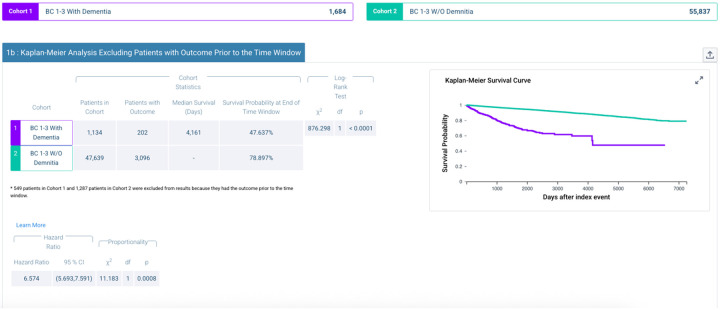
Kaplan Meier Survival Curve by Dementia Status

Kaplan–Meier Survival Analysis. Survival analysis (Fig. 2) further highlighted the heightened fall risk in patients with dementia. The median survival time to first fall in the dementia cohort was 4,161 days, with only 47.6% of patients remaining fall-free at the end of the follow-up window, compared with 78.9% in the non-dementia cohort. The log-rank test revealed a highly significant difference between survival curves (χ^2^ = 876.3, df = 1, p < 0.0001). The hazard of falling was more than six times higher among breast cancer survivors with dementia (HR = 6.57, 95% CI 5.69–7.59, p = 0.0008).

Number of Fall Instances. When the frequency of fall episodes was evaluated, dementia patients averaged slightly more fall events than those without dementia (2.44 ± 8.20 vs 2.04 ± 5.05). However, this difference was not statistically significant (t = 1.05, df = 3,296, p = 0.29). Median fall count was one in both cohorts, suggesting that while dementia substantially increases the likelihood and timing of falls, the total number of fall episodes per patient among those who do fall does not differ meaningfully by dementia status.

### Cox Proportional Hazards Analysis

A multivariable Cox proportional hazards model was used to examine predictors of fall-related outcomes among older breast cancer survivors. Dementia status was independently associated with a significantly higher hazard of falls (HR = 1.43, 95% CI: 1.25–1.63, p < 0.001) after adjustment for demographic, clinical, and functional covariates (Table 7).

Age and Demographics. Each one-year increase in age at index was associated with a 5.4% increase in fall risk (HR = 1.05, 95% CI: 1.05–1.06, p < 0.001). Race and ethnicity were significant predictors: Black or African American patients had a 30% higher hazard of falls compared with White patients (HR = 1.31, 95% CI: 1.20–1.42, p < 0.001), while Hispanic or Latino patients had the highest relative risk (HR = 1.77, 95% CI: 1.55–2.03, p < 0.0001).

Frailty and Musculoskeletal Conditions. Among frailty-related variables, osteoporosis (HR = 1.48, 95% CI: 1.34–1.62, p < 0.001) and cachexia (HR = 2.44, 95% CI: 1.45–4.08, p = 0.001) were strong independent predictors of falls. Sarcopenia and muscle wasting were not statistically significant (p = 0.44 and p = 0.71, respectively).

Neurological Disorders. Polyneuropathy was associated with a 57% higher hazard of falls (HR = 1.58, 95% CI: 1.34–1.85, p < 0.001), while Parkinson’s disease (HR = 1.50, 95% CI: 1.12–2.01, p = 0.007) and sequelae of cerebral infarction (HR = 1.53, 95% CI: 1.15–2.03, p = 0.003) were also significant. Unsteadiness on feet (HR = 1.35, 95% CI: 1.08–1.71, p = 0.010) and other gait abnormalities (HR = 1.42, 95% CI: 1.22–1.66, p < 0.001) further contributed to elevated fall risk.

Psychiatric Conditions. Both depressive episodes (HR = 1.78, 95% CI: 1.60–1.98, p < 0.001) and recurrent major depressive disorder (HR = 1.50, 95% CI: 1.26–1.79, p < 0.001) were independently associated with increased fall risk. Delirium and mild cognitive impairment were not significant predictors (p = 0.51 and p = 0.28, respectively).

Comorbidities. Heart failure was a significant predictor of falls (HR = 1.36, 95% CI: 1.20–1.53, p < 0.001), whereas malnutrition, pressure ulcers, and visual loss did not reach statistical significance (all p > 0.12).

Medication Burden. Long-term drug therapy, used as a proxy for polypharmacy (ICD-10-CM Z79), was among the strongest predictors, associated with more than a twofold increased hazard of falls (HR = 2.62, 95% CI: 2.41–2.84, p < 0.001).

## DISCUSSION

This large real-world cohort study provides new insights into the relationship between dementia and fall risk among older breast cancer survivors. We observed that survivors with dementia were more than twice as likely to experience a fall compared with survivors without dementia, and this increased risk persisted after controlling for a comprehensive set of demographics, clinical and treatment-related factors. The absolute risk difference of 11.3 percentage points translates into a substantial burden of fall-related events in this population. These findings align with broader geriatric literature showing that cognitive impairment markedly increases the likelihood of falls: older adults with dementia fall two to three times more often than cognitively healthy peers, and between 60–80% of people with dementia fall annually^11^. Our results extend these observations by showing that dementia remains an independent predictor of falls even after accounting for frailty, neurological disorders, psychiatric conditions and polypharmacy, suggesting a distinctive vulnerability among cognitively impaired cancer survivors.

Several covariates emerged as important contributors to fall risk. Increasing age at cancer diagnosis was associated with a higher hazard of falling, consistent with evidence that falls affect more than thirty percent of adults aged sixty-five years and older^15^. Race and ethnicity were also significant predictors: Black and Hispanic survivors faced greater hazards compared with White survivors^16^. Frailty-related conditions such as osteoporosis and cachexia, neurologic disorders including polyneuropathy, Parkinson disease and sequelae of cerebral infarction, and psychiatric disorders such as depressive episodes all conferred elevated fall risk^17–20^. Notably, long-term use of medications a proxy for polypharmacy was one of the strongest predictors, more than doubling the hazard of falling^21^. These findings highlight the multifactorial nature of falls, which are influenced by interactions between neuromuscular function, cognition, comorbidity burden, medication effects and sociodemographic factors. They also underscore the importance of comprehensive geriatric assessment in oncology practice, with attention to medication management and mental health.

Our findings have several implications for clinical care and survivorship planning. First, they emphasize the need for proactive fall-prevention strategies tailored to older breast cancer survivors with dementia. Interventions such as balance and strength training, home safety assessments, medication review and cognitive support should be integrated into survivorship care plans. Second, the strong association between polypharmacy and falls supports efforts to deprescribe non-essential medications and to coordinate care across oncology, primary care and geriatrics. Third, the elevated risk among Black and Hispanic survivors signals potential disparities in access to supportive services or differences in comorbidity profiles; culturally informed interventions and equitable access to fall-prevention programs are needed. Finally, because older adults with dementia fall frequently, clinicians should consider routine cognitive screening for breast cancer survivors and engage caregivers in fall-prevention education. Future research should examine the efficacy of multifaceted interventions in this population, explore mechanisms linking cognitive impairment and falls, and investigate how cancer treatments interact with dementia-related neuropathology to influence mobility and balance.

Strength. The study’s design affords several notable strengths that enhance confidence in its findings. Leveraging the TriNetX platform allowed us to assemble one of the largest real-world cohorts of older breast cancer survivors with and without dementia to date. Because TriNetX aggregates records from over 100 participating health systems, the resulting sample encompasses diverse patient populations and practice settings, which improves the generalizability of the results. Moreover, the use of rigorous statistical methods including one-to-one propensity score matching and adjustments for a comprehensive array of demographic, clinical and medication variables which helped minimize confounding and ensured that the observed associations were not driven by imbalances between cohorts. Our precise outcome definitions, which incorporated multiple International Classification of Diseases codes (ICD- code) for fall events, captured a wide spectrum of falls from single incidents to recurrent episodes and fall-related injuries. This approach aligns with broader evidence that cognitive impairment markedly increases fall risk: older adults with dementia fall two to three times more frequently than cognitively healthy peers, and between sixty and eighty percent of individuals with dementia fall each year. These methodological strengths yield robust, broadly applicable insights into fall risk among this medically complex population. The Kaplan Meier survival analysis provided an unadjusted age-based estimate of fall-free survival, illustrating the cumulative incidence of falls over time before accounting for other covariates in multivariable models.

Limitation. Several limitations should be considered when interpreting the results. The retrospective nature of electronic health record analysis precludes causal inference and relies on accurate coding. Falls that did not result in medical attention were not captured, potentially underestimating the true incidence of fall events. Although we adjusted for many potential confounders, residual confounding may persist because data on certain factors such as the severity and type of dementia, detailed cancer treatments, functional status or living environment were unavailable. The “unspecified dementia” category used here includes heterogeneous etiologies, which prevented us from examining how different dementia subtypes might influence fall risk. Additionally, while TriNetX provides a large and diverse sample, its composition may not fully represent populations outside North America, and coding practices may vary across contributing institutions, introducing some misclassification bias. These factors highlight the need for future prospective studies with more granular data to confirm and extend the current findings.

## Figures and Tables

**Figure 1:**
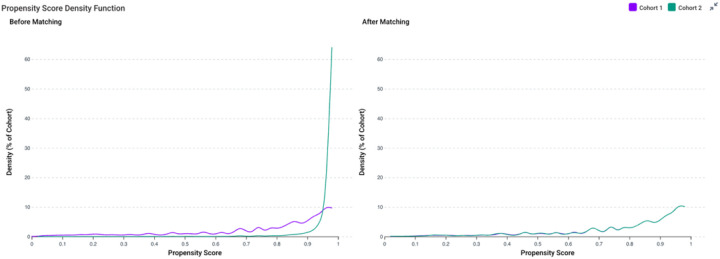
Propensity score distributions before and after matching Copy/right of the graph to TriNetX Platform

**Table 1:** Covariates and ICD-10-CM Codes Used in the Study

Category	Variable	ICD-10-CM Code(s)	Definition / Description
**Frailty and Musculoskeletal Conditions**	Osteoporosis	M81.0	Age-related osteoporosis without current pathological fracture
	Sarcopenia	M62.84	Age-related loss of skeletal muscle mass and strength
	Cachexia	R64	Wasting syndrome characterized by weight loss and muscle atrophy
	Muscle wasting / Atrophy	M62.5	Muscle wasting and atrophy, not elsewhere classified
**Neurological Disorders**	Polyneuropathy, unspecified	G62.9	Peripheral nerve disorder of unspecified cause
	Drug-induced polyneuropathy	G62.0	Neuropathy secondary to medication or toxin exposure
	Parkinson’s disease	G20	Idiopathic Parkinson’s disease
	Sequelae of cerebral infarction	I69.3	Residual effects following stroke or cerebral infarction
	Unsteadiness on feet	R26.81	Difficulty maintaining balance during ambulation
	Other abnormalities of gait and mobility	R26.89	Gait disturbance not classified elsewhere
**Psychiatric Conditions**	Major depressive disorder, recurrent	F33	Recurrent episodes of major depression
	Depressive episode	F32	Single episode of depression
**Comorbidities**	Heart failure	I50	Heart failure of any type (systolic, diastolic, or combined)
	Malnutrition, unspecified	E46	Unspecified protein-calorie malnutrition
	Severe protein-calorie malnutrition	E43	Severe form of protein-calorie deficiency
	Pressure ulcer	L89	Pressure-induced skin ulcer
**Medication Burden / Polypharmacy**	Long-term (current) drug therapy	Z79	Long-term use of medications as a proxy for polypharmacy

**Table 2:** Baseline Demographic Characteristics of Breast Cancer Survivors With and Without Dementia

Demographics	Term / Code	Dementia Cohort (n = 1,683)	No Dementia Cohort (n = 48,926)	P-Value	Std. Diff.
Age	Current Age, mean ± SD	84.3 ± 6.44	76.3 ± 7.54	< 0.0001	1.1432
	Age at Index, mean ± SD	79.9 ± 7.4	67.2 ± 8.84	< 0.0001	1.5523
Ethnicity	Not Hispanic or Latino	70% (n = 1,174)	50% (n = 24,562)	< 0.0001	0.4073
	Unknown Ethnicity	27% (n = 446)	46% (n = 22,487)	< 0.0001	0.4134
	Hispanic or Latino	3% (n = 63)	4% (n = 1,877)	0.8449	0.0049
Race	White	66% (n = 1,104)	59% (n = 28,657)	< 0.0001	0.1452
	Black or African American	25% (n = 414)	11% (n = 5,618)	< 0.0001	0.3462
	Asian	5% (n = 86)	4% (n = 2,083)	0.0896	0.0404
	Unknown Race	2% (n = 35)	18% (n = 9,038)	< 0.0001	0.5607
	Other Race	2% (n = 27)	6% (n = 3,140)	< 0.0001	0.2472
	Native Hawaiian / Pacific Islander	1% (n = 13)	1% (n = 344)	0.7383	0.0081
	American Indian / Alaska Native	1% (n = 10)	< 1% (n = 46)	< 0.0001	0.0855

**Table 3:** Baseline Fall-Related Diagnoses in Breast Cancer Patients With and Without Dementia

Term / Code	Dementia Cohort 1 (n = 1,683)	No Dementia Cohort 2 (n = 48,926)	P-Value	Std. Diff.
W19 – Unspecified fall	26% (n = 434)	2% (n = 1,038)	< 0.0001	0.7266
W19.XXXA – Unspecified fall, initial encounter	25% (n = 415)	2% (n = 980)	< 0.0001	0.7069
Z91.81 – History of falling	15% (n = 259)	1% (n = 403)	< 0.0001	0.5537
R29.6 – Repeated falls	11% (n = 180)	0% (n = 191)	< 0.0001	0.4622
T14 – Injury of unspecified body region	8% (n = 141)	1% (n = 576)	< 0.0001	0.3425

**Table 4:** Baseline Characteristics Before and After Propensity Score Matching

Variable	Category	Before Matching: Dementia Cohort 1(n = 1,683)	Before Matching: No Dementia Cohort 2(n = 48,926)	P-Value	Std. Diff.	After Matching: Dementia Cohort 1(n = 1,602)	After Matching: No Dementia Cohort 2(n = 1,602)	Std. Diff.	P-Value
**Age (years)**	Current Age (mean ± SD)	84.3 ± 6.44	76.3 ± 7.54	< 0.0001	1.1432	84.1 ± 6.50	84.1 ± 6.56	0.984	0.0007
	Age at Index (mean ± SD)	79.9 ± 7.40	67.2 ± 8.84	< 0.0001	1.5523	79.5 ± 7.39	79.5 ± 7.52	0.986	0.0006
**Ethnicity**	Not Hispanic or Latino	1,174 (69.76%)	24,562 (50.20%)	< 0.0001	0.4073	1,116 (69.66%)	1,153 (71.97%)	0.150	0.0508
	Unknown Ethnicity	446 (26.50%)	22,447 (45.96%)	< 0.0001	0.4134	426 (26.59%)	393 (24.53%)	0.181	0.0472
	Hispanic or Latino	63 (3.74%)	1,877 (3.84%)	0.8449	0.0049	60 (3.75%)	56 (3.50%)	0.705	0.0134
**Race**	White	1,104 (65.60%)	28,657 (58.57%)	< 0.0001	0.1452	1,051 (65.61%)	1,048 (65.42%)	0.911	0.0039
	Black or African American	414 (24.60%)	5,618 (11.48%)	< 0.0001	0.3462	390 (24.35%)	408 (25.47%)	0.462	0.0260
	Asian	86 (5.11%)	2,083 (4.26%)	0.0896	0.0404	83 (5.18%)	85 (5.31%)	0.874	0.0056
	Unknown Race	35 (2.08%)	9,038 (18.47%)	< 0.0001	0.5607	35 (2.19%)	27 (1.69%)	0.304	0.0363
	Other Race	27 (1.60%)	3,140 (6.42%)	< 0.0001	0.2472	27 (1.69%)	23 (1.44%)	0.568	0.0201
	Native Hawaiian / Pacific Islander	13 (0.77%)	344 (0.70%)	0.7383	0.0081	13 (0.81%)	10 (0.62%)	0.530	0.0222
	American Indian / Alaska Native	10 (0.59%)	46 (0.09%)	< 0.0001	0.0855	10 (0.62%)	10 (0.62%)	1.000	< 0.0001

**Table 5:** Baseline Fall-Related Diagnoses Before and After Propensity Score Matching

Variable Fall-Related Diagnoses	Before Matching: Dementia Cohort 1 (n = 1,683)	Before Matching: No Dementia Cohort 2 (n = 48,926)	P-Value	Std. Diff.	After Matching: Dementia Cohort 1 (n = 1,602)	After Matching: No Dementia Cohort 2 (n = 1,602)	P-Value	Std. Diff.
Unspecified Fall	434 (25.79%)	1,038 (2.12%)	< 0.0001	0.7266	366 (22.85%)	370 (23.10%)	0.8666	0.0059
Unspecified Fall, Initial Encounter	415 (24.66%)	980 (2.00%)	< 0.0001	0.7069	349 (21.79%)	356 (22.22%)	0.7653	0.0105
History of Falling	259 (15.39%)	403 (0.82%)	< 0.0001	0.5537	201 (12.55%)	180 (11.24%)	0.2517	0.0405
Repeated Falls	180 (10.70%)	191 (0.39%)	< 0.0001	0.4622	134 (8.37%)	112 (6.99%)	0.1443	0.0516
Injury of Unspecified Body Region	141 (8.38%)	576 (1.18%)	< 0.0001	0.3425	119 (7.43%)	127 (7.93%)	0.5955	0.0188

**Table 6:** Measures of Association (Excluding Patients with Outcome Prior to the Time Window)

Cohort	Patients in Cohort	Patients with Outcome	Risk (%)
BC 1–3 With Dementia	1,134	202	**17.81%**
BC 1–3 Without Dementia	47,639	3,096	**6.50%**

Measure	Estimate	95% CI	z	p
**Risk Difference**	11.31%	(9.08%, 13.55%)	14.996	<0.0001
**Risk Ratio**	2.74	(2.41, 3.12)	N/A	N/A
**Odds Ratio**	3.12	(2.67, 3.65)	N/A	N/A

549 patients in Cohort 1 and 1,287 patients in Cohort 2 were excluded for having outcome prior to time window.

**Table 7:** Multivariable Cox Proportional Hazards Model for Predictors of Fall-Related Outcomes Among Older Breast Cancer Survivors

Cox Model Results								
Category	Code	Covariate	Hazard Ratio	Coefficient	Standard Error	z	P >|z|	95% Confidence Interval
		Cohort 1 or Cohort 2 Membership	1.430	0.358	0.068	5.278	< 0.001	(1.252, 1.634)
Age	AI	Age at Index	1.054	0.053	0.002	27.837	< 0.001	(1.051, 1.058)
Race	2054–5	Black or African American	1.306	0.267	0.044	6.109	< 0.001	(1.199, 1.423)
Ethnicity	2135–2	Hispanic or Latino	1.773	0.573	0.068	8.364	< 0.001	(1.551, 2.028)
Frailty and Musculoskeletal Conditions	M81.0	Osteoporosis	1.476	0.389	0.049	7.975	< 0.001	(1.341, 1.624)
	M62.84	Sarcopenia	1.467	0.383	0.499	0.768	0.443	(0.551, 3.905)
	R64	Cachexia	2.435	0.890	0.263	3.383	0.001	(1.454, 4.077)
	M62.5	Muscle wasting	0.922	−0.082	0.219	−0.372	0.710	(0.600, 1.417)
Neurological Disorders	G62.9	Polyneuropathy, unspecified	1.575	0.454	0.082	5.546	< 0.001	(1.341, 1.849)
	G62.0	Drug-induced polyneuropathy	0.654	−0.424	0.262	−1.619	0.105	(0.391, 1.093)
	G20	Parkinson's disease	1.498	0.404	0.150	2.698	0.007	(1.117, 2.008)
	I69.3	Sequelae of cerebral infarction	1.527	0.423	0.145	2.928	0.003	(1.150, 2.027)
	R26.81	Unsteadiness on feet	1.354	0.303	0.117	2.586	0.010	(1.076, 1.705)
	R26.89	Other abnormalities of gait and mobility	1.418	0.349	0.079	4.418	< 0.001	(1.215, 1.656)
**Psychiatric Conditions**	F32	Depressive episode	1.779	0.576	0.054	10.749	< 0.001	(1.602, 1.977)
	F33	Major depressive disorder, recurrent	1.501	0.406	0.090	4.521	< 0.001	(1.259, 1.790)
	F05	Delirium	0.887	−0.120	0.181	−0.662	0.508	(0.622, 1.265)
	G31.84	Mild cognitive impairment	1.129	0.122	0.112	1.086	0.277	(0.907, 1.407)
**Comorbidities**	I50	Heart failure	1.355	0.304	0.062	4.877	< 0.001	(1.199, 1.531)
	E46	Unspecified protein-calorie malnutrition	1.047	0.046	0.165	0.277	0.782	(0.757, 1.448)
	E43	Unspecified severe protein-calorie malnutrition	0.869	−0.140	0.221	−0.634	0.526	(0.564, 1.340)
	L89	Pressure ulcer	0.722	−0.326	0.213	−1.531	0.126	(0.476, 1.096)
	H54.7	Unspecified visual loss	1.129	0.121	0.155	0.782	0.434	(0.833, 1.531)
**Medication Burden / Polypharmacy**	Z79	Long term (current) drug therapy	2.615	0.961	0.043	22.607	0.001	(2.406, 2.842)

## Data Availability

The data that support the findings of this study are available upon reasonable request. Interested researchers can obtain access to the data by submitting a formal request to the corresponding author at Asmaa.namoos@vcuhealth.org. The data is not publicly available due to privacy or ethical restrictions.
